# Multiplex CRISPR-Cas9 mutagenesis of the phytochrome gene family in *Physcomitrium (Physcomitrella) patens*

**DOI:** 10.1007/s11103-020-01103-x

**Published:** 2020-12-21

**Authors:** Silvia Trogu, Anna Lena Ermert, Fabian Stahl, Fabien Nogué, Tanja Gans, Jon Hughes

**Affiliations:** 1 Institute for Plant Physiology, Justus Liebig University, Senckenbergstrasse 3, 35390 Giessen, Germany; 2 Institut Jean-Pierre Bourgin, INRAE, AgroParisTech, Université Paris-Saclay, 78000 Versailles, France

**Keywords:** CRISPR-Cas9, Lower plants, Phytochrome, Multiplex gene editing, Homologous recombination, Gravitropism

## Abstract

**Key message:**

We mutated all seven *Physcomitrium (Physcomitrella) patens* phytochrome genes using highly-efficient CRISPR-Cas9 procedures. We thereby identified phy5a as the phytochrome primarily responsible for inhibiting gravitropism, proving the utility of the mutant library.

**Abstract:**

The CRISPR-Cas9 system is a powerful tool for genome editing. Here we report highly-efficient multiplex CRISPR-Cas9 editing of the seven-member phytochrome gene family in the model bryophyte *Physcomitrium (Physcomitrella) patens*. Based on the co-delivery of an improved Cas9 plasmid with multiple sgRNA plasmids and an efficient screening procedure to identify high-order multiple mutants prior to sequencing, we demonstrate successful targeting of all seven *PHY* genes in a single transfection. We investigated further aspects of the CRISPR methodology in Physcomitrella, including the significance of spacing between paired sgRNA targets and the efficacy of NHEJ and HDR in repairing the chromosome when excising a complete locus. As proof-of-principle, we show that the septuple *phy*^*−*^ mutant remains gravitropic in light, in line with expectations, and on the basis of data from lower order multiplex knockouts conclude that phy5a is the principal phytochrome responsible for inhibiting gravitropism in light. We expect, therefore, that this mutant collection will be valuable for further studies of phytochrome function and that the methods we describe will allow similar approaches to revealing specific functions in other gene families.

**Supplementary information:**

The online version of this article (10.1007/s11103-020-01103-x) contains supplementary material, which is available to authorized users.

## Introduction

Originally identified as part of the prokaryotic adaptive immunity providing resistance to phage, virus and plasmid infection, the CRISPR-Cas9 nuclease system has proved to be a powerful tool for genome manipulation even in eukaryotes, and has rapidly become the most widely used technology for genome editing (Jinek et al. 2012; Knott and Doudna 2018). This programmable restriction enzyme system comprises two components: a 20 bp single guide RNA (sgRNA) to target a specific locus, and the Cas9 endonuclease (Anders et al. 2014, 2015; Jinek et al. 2014) for the induction of a double strand break (DSB) at the target site. The only prerequisite for cleavage is the presence of a protospacer adjacent motif (PAM) downstream of the target. Cas9 then cleaves at a specific point usually located three or four nucleotides upstream of the PAM (Jinek et al. 2012).

CRISPR's mutagenic ability derives from the action of endogenous DNA repair pathways after the DSB has taken place. DSBs are usually repaired by the non-homologous end-joining (NHEJ) machinery that often creates random insertions or deletions (indels) at the target site. NHEJ is especially efficient when the sequence near the DSB shows microhomology (thus microhomology‐mediated end joining (MMEJ)). If an indel occurs within a coding region, function is often lost on account of the shifted translational reading frame. Even complete loci can be excised if appropriately-spaced dual sgRNAs are employed. On the other hand, if an exogenous donor DNA flanked by sequences homologous to the target site is provided, homology-dependent repair (HDR) may occur instead, in which case, a precisely customized gene can be generated with the help of an appropriate repair sequence.

In contrast to other prokaryotic and eukaryotic groups, seed plants (spermatophytes) show near-zero rates of homologous recombination (HR) (Horvath et al. 2016), NHEJ repair predominating in somatic cells (Puchta 2004). As gene targeting has huge benefits in breeding strategies, the development of HR-based methods in plants is of great importance. In this context, we have studied gene targeting in Physcomitrella*,* a bryophyte that naturally exhibits high frequencies of HR—the discovery of which enabled high-efficiency gene targeting in plants for the first time (Schaefer and Zrÿd 1997). Indeed, Physcomitrella represents a model system particularly suited for fundamental studies of plant biology, its utility being reinforced by its ease of genetic transformation and regeneration, small size and predominantly haploid life cycle. The complete genome sequence is publicly available (Rensing et al. 2008).

To date, CRISPR has been successfully applied in many plant species to edit single or multiple genes (Najera et al. 2019). The ability to create multiplex knockouts is especially useful in studying gene families or other cases of redundant function (Lopez-Obando et al. 2016). This is particularly significant in the case of Physcomitrella because it has undergone two rounds of whole-genome duplications during its evolution, which has contributed to the expansion of several gene families (Li et al. 2015). Moreover, the high rate of HR in Physcomitrella (Schaefer and Zrÿd 1997) might provide a particularly fertile field for CRISPR-induced gene modification in plants. Established transfection and regeneration protocols for Physcomitrella (Cove et al. 2009a, b, c) are appropriate for CRISPR applications, and indeed, both Cas9-mediated gene knock-out (KO) (with NHEJ or MMEJ) and knock-in (with HDR) have proven to be successful, including the capability to target multiple genes in a single transfection experiment (multiplexing) (Collonnier et al. 2017; Lopez-Obando et al. 2016; Nomura et al. 2016). More recently, a modular CRISPR‐Cas9 vector system has been developed (Mallett et al. 2019) and multiplexing has also been demonstrated using the Cas12a (Cpf1) (Pu et al. 2019), thus expanding the Physcomitrella genetic toolkit.

A collection of multiplex KO lines allows gene functions to be identified even in the face of functional redundancy. An interesting and important case in this regard is the phytochrome (*PHY*) gene family. Phytochromes are red/far-red photochromic photoreceptors that act as master developmental switches, regulating the transcription of thousands of genes in all plants. Intriguingly, phytochrome is also able to steer the growth direction of the filaments in bryophytes, although that cannot be achieved via gene regulation as the directional information is lost in transcription/translation (Hughes 2013). Unfortunately for genetic analysis of these functions, however, the Physcomitrella genome (Lang et al. 2018; Rensing et al. 2008; Zimmer et al. 2013) encodes no less than seven phytochrome genes (*PHY1* to *PHY4* and *PHY5a* to *PHY5c*), more than known in any other species to-date, reflecting following whole genome duplication and perhaps also an evolutionary strategy to enhance acclimation in diverse light environments (Hughes 2013). As the different phytochromes have both specific and overlapping functions, the efficacy of using multiplex CRISPR in this context is rather clear. On the other hand, the 7 PHY genes show extensive sequence similarities reaching an identity of 80% higher than any of the seed-plant phytochromes (Li et al. 2015). CRISPR-based targeting of the phytochrome gene family in Physcomitrella is thus a challenging project.

In the present study, we describe CRISPR-based multiplex gene editing in Physcomitrella, focusing on the phytochrome gene family, successfully mutating all seven PHY genes in a single experiment. We have also developed a novel and efficient method for multiple indel screening and tested Cas9 efficiency in excising a complete gene or a small fragment either with or without HDR. In the process, we have generated a mutant collection for the community that will allow systematic analysis of phytochrome function in Physcomitrella*.* We demonstrate the efficacy of this by showing that suppression of gravitropism is lost completely in the septuple *phy*^−^ mutant and that phytochrome 5a is the photoreceptor principally responsible.

## Materials and methods

### Design of sgRNAs and repair constructs

The coding sequences of the 7 *PHYTOCHROME* genes, including up- and downstream flanking sequences, were used to search for appropriate CRISPR RNA (crRNA) targets using the CRISPOR server (http://crispor.tefor.net/, Haeussler et al. 2016) with the Physcomitrella V11 genome sequence. crRNAs with high specificity scores and few potential off-target sequences were selected for cloning (Supplementary Information, Table S2). Each sgRNA and HR fragment was then cloned into the Gateway donor vector pDONR207, transferred to the pENTRsgRNA vector and the final constructs harvested from *E. coli* by alkaline lysis and purified by PEG/NaCl precipitation (Sambrook et al. 1989).

**Fig. 1:**
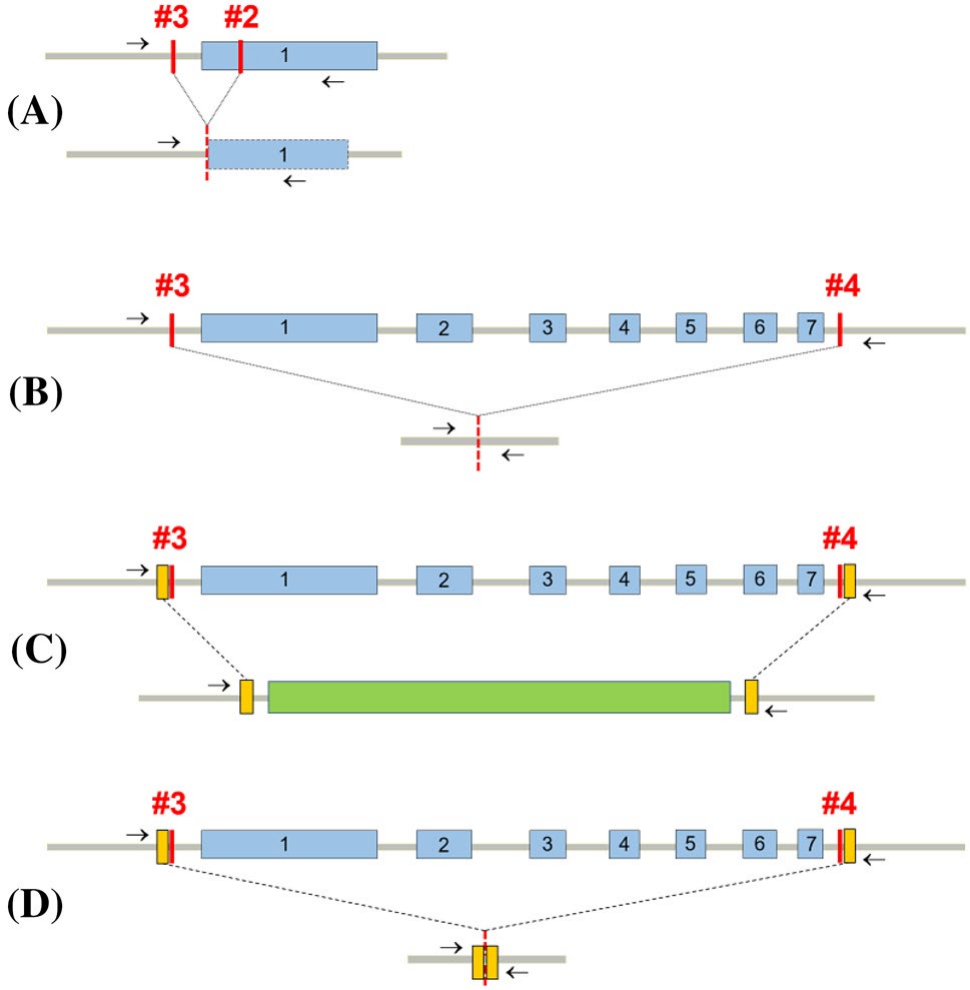
The *PHY4* genomic locus with different Cas9-mediated genome editing approaches. **a** Excision of a ~ 480 bp fragment between the 5′UTR region and the first exon. **b** Excision of the entire ~ 6 kbp *PHY4* locus via NHEJ. **c**, **d** Replacement of the *PHY4* locus via HDR with and without the hygromycin resistance cassette, respectively. The exons are shown in blue and numbered. The sgRNAs are numbered #2–#4 and the PCR primers shown as small arrows. The hygromycin resistance cassette is shown in green. The homologous regions for HDR are shown in gold

Dual-cut experiments exploiting NHEJ and HDR (Tables 2 and 3) (Ermert et al. 2019) are illustrated in Fig. 1.

### Moss culture, protoplast isolation and transfection

*Physcomitrium (Physcomitrella) patens patens* (Gransden strain) was grown in 16 h white light (50–80 µmol/m^2^ s photosynthetically active radiation) and 8 h dark at 21 °C. Protonemata were cultured for 6 days on PpNH4 medium and harvested for protoplast isolation using Driselase (Sigma) and transfection as described (Cove et al. 2009a, c). Each transfection (except Tables 1e, f and 3a) included 8 µg of the pAct-Cas9 plasmid, the pBHRF plasmid containing a hygromycin resistance cassette for transient selection, and the appropriate sgRNA plasmids (Lopez-Obando et al. 2016). The pAL114 plasmid was constructed by cloning the blunted BanII-digested 35S::hygromycin resistance cassette from pBHRF into blunted ApaI-digested pActCas9 (Lopez-Obando et al. 2016). In transfections targeting all seven *PHY* genes (Table 1e), the mixture comprised the pAL114 plasmid and all sgRNA-plasmids, while in the retransfection experiment (Table 1f) the quintuple KO mutant (Table 1d) was transfected with the pSCOE-fcoCas9 plasmid (Nomura et al. 2016) and sgRNA-plasmids targeting *PHY5a* and *PHY5b*. In the dual-cut transfections (Tables 2 and 3, Fig. 1), two sgRNA plasmids, and in the case of HDR a plasmid carrying appropriate PHY4 sequences with or without a stably-selectable marker between the gene-flanking homologous regions, were included in the mixture. Protoplasts were transfected with a total of 20–25 µg of plasmid DNA. Protoplasts were allowed to regenerate on cellophane-overlaid PRMB plates for 1 week. Filaments were then transferred to antibiotic-supplemented medium without mannitol for selection of transfected cells. After another week, regenerants were placed on BCE225 plates without selection and grown further for 2 weeks. Finally, individual regenerants were transferred to wells in microtiter plates. After 4–6 months the loss of transient antibiotic resistance was verified through lethal reselection.

**Table 1:** Multiplex *PHY* mutagenesis using single sgRNAs

Transfection	Targets	Total mutants identified
a	2-fold: *PHY2* + *PHY4*	26/75 (35%)
b	2-fold: *PHY5b* + *PHY5c*	38/48 (79%)
c	4-fold: *PHY1-4*	12/60 (20%)
d	6-fold: *PHY1-4* + *PHY5b-c*	28/74 (38%)
e	7-fold: *PHY1-5c*	80/85 (94%)
f	5 + 2-fold: *PHY5a* + *PHY5b* in a quintuple mutant background	47/53 (89%)

**Table 2:** NHEJ-mediated *PHY4* editing with dual sgRNAs

Transfection	Targets	Mutants identified
a	Excision of 480 bp fragment	2/39 (5.1%)
b	Excision of entire ~ 6 kbp locus	11/44 (25%)

**Table 3:** HDR-mediated ~ 6 kbp *PHY4* excision with dual sgRNAs

Transfection	HDR construct	Mutants identified
a	With hyg^R^ insertion	5/23 (22%)
b	Control (no insert)	1/63 (1.6%)

### High throughput DNA extraction

In initial work, gDNA was extracted using a Mixer Mill (MM300; Retsch, Haan, Germany) followed by CTAB (cetyltrimethylammonium bromide) precipitation according to established methods. Later, a high-throughput procedure using 96-well microtitre plates exploiting cellulase rather than mechanical disruption of the cell wall was developed. Thereby, approximately 20 mg fresh weight of actively-growing filaments were treated enzymatically in 2% (w/v) Driselase in 8% mannitol for 45 min at 25–30 °C, then centrifuged at 3500×*g* for 10 min at 20 °C. After discarding the supernatants, 250 µl of extraction buffer (2% CTAB, 100 mM Tris/HCl pH 8.0, 20 mM EDTA pH 8.0, 1.4 M NaCl, 1% w/v PVP and freshly added 50 mM DTT) was added to each well, the plate then shaken briefly, incubated at 65 °C for 5 min and then centrifuged at 3500×*g* for 10 min at 20 °C. 200 µl of each supernatant was transferred to a fresh plate and the gDNA precipitated by adding 1 volume of isopropanol and mixing. Following centrifugation at 3500×*g* for 25 min at 20 °C, the supernatants were discarded and the CTAB/DNA pellets washed in 70% ethanol then allowed to dry prior to uptake in 10 µl TE buffer.

### Knockout and off-target screening

**Fig. 2:**
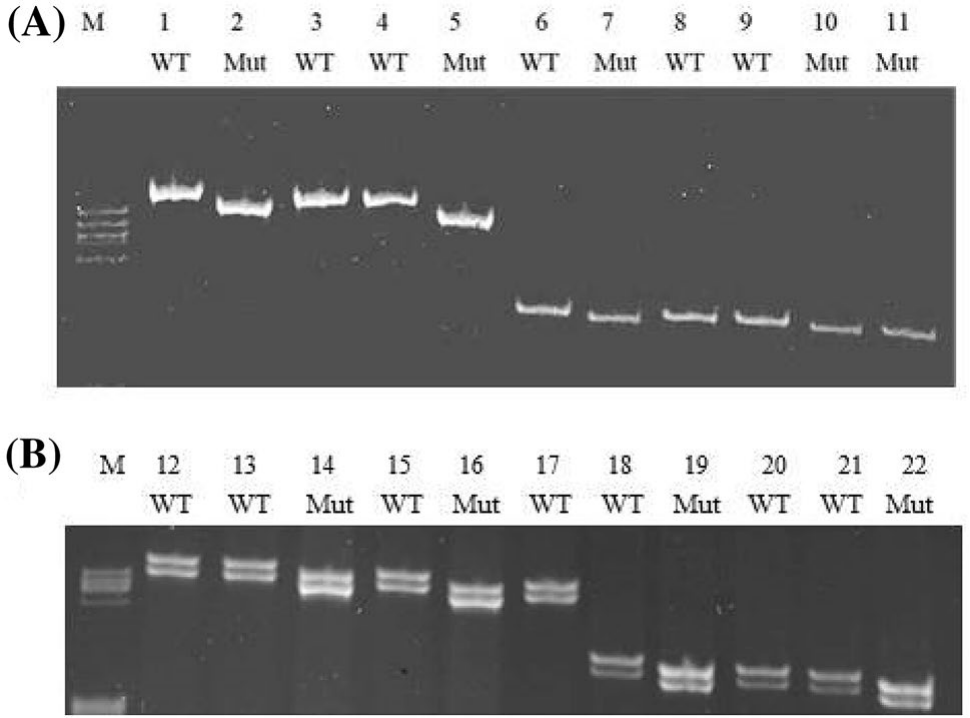
Identification of Cas9-mediated indels in high-resolution PAGE Short DNA fragments (~ 100 bp) surrounding each target site were amplified and separated on 15% polyacrylamide gels. **a** Lanes 1–5 and 6–11 show *PHY5a* and *PHY2* products, respectively, from different lines amplified with Phusion High-Fidelity DNA Polymerase. Lanes 2 & 5 and 7, 10 & 11 show 2 and 1 bp deletions, respectively*.* **b** Lanes 12–17 and 18–22 show the same extracts amplified with Taq DNA Polymerase. Lanes 14 & 16 and 19 & 22 show 2 & 1 bp deletions, respectively

10 µl PCR reactions were performed using 1 µl of extracted DNA as template and Phusion High-Fidelity DNA Polymerase or Taq DNA Polymerase (New England Biolabs). ~ 100 bp fragments flanking each target site were amplified using appropriate gene-specific primers and separated on 15 cm 15% TBE/PAGE gels, allowing even 1 bp indels to be detected (Fig. 2). Appropriate products were then sequenced (Supplementary Information, Table S1). Sequences were aligned using Clustal and inspected for mutations around the PAM sequence.

Potential mutants produced in dual-cut transfections were tested by PCR using primers flanking the two target sites as previously described (Ermert et al. 2019).

Off-target mutants for each sgRNA used in the multiplex editing (Table 1a–f) were sought by PCR/PAGE directed to all 8 CRISPOR-predicted off-target sites in the six- and sevenfold mutants (Supplementary Information, Table S3). None was found. Coincidentally, the only predicted off-target site with less than four mismatches was that of *PHY5c*-sgRNA in the *PHY5b* gene, in a region that we had sequenced many times while screening putative *phy5b*^*−*^ mutants.

### Phenotypic analysis

The effect of light on gravitropism was assayed as described (Lamparter et al. 1996) with at least two different mutant lines of each genotype in several independent experiments. Homogenized protonemata were plated on cellophane-covered agar medium and grown for 7–10 days. Approximately 2 mm slices were then placed on vertically-orientated square Perti dishes and grown for 6 days in darkness to generate uniform caulonemata. The plates were then rotated by 90° and either left in darkness or irradiated with 5 µmol/m^2^ s of 660 nm red light from behind the plate for 3 days. The filaments were photographed using a zoom macroscope (Macro Z16 with DFX 500 CCD camera; Leica Microsystems, Wetzlar, Germany) and the tip orientations assayed using ImageJ (1.52d).

## Results

### Screening

Screening the indel complement of 7 loci in numerous lines is a daunting exercise. Although we routinely extracted gDNA from filaments using mixer-mill disruption followed by CTAB extraction/precipitation, methods offering higher through-put were clearly desirable. We thus developed a procedure appropriate for 96-well plates in which the filament cell walls were degraded enzymatically, allowing the nuclear material to be released into the medium without mechanical intervention. gDNA was subsequently extracted using CTAB. We also developed a pre-screening procedure in which indels in each locus could be identified prior to sequencing using PCR. By amplifying only a small ~ 100 bp region flanking the target site and analyzing the products on ~ 20 cm 15% polyacrylamide gels with appropriate resolution, we were able to detect even 1 bp indels confidently (Fig. 2). Whereas the expected single-band products were obtained using error-checking polymerases, we were surprised to find that Taq polymerase generated double-band products of almost equal strength with a mobility difference corresponding to 2 bp (Fig. 2b).

### Multiplex phytochrome gene editing

**Fig. 3:**
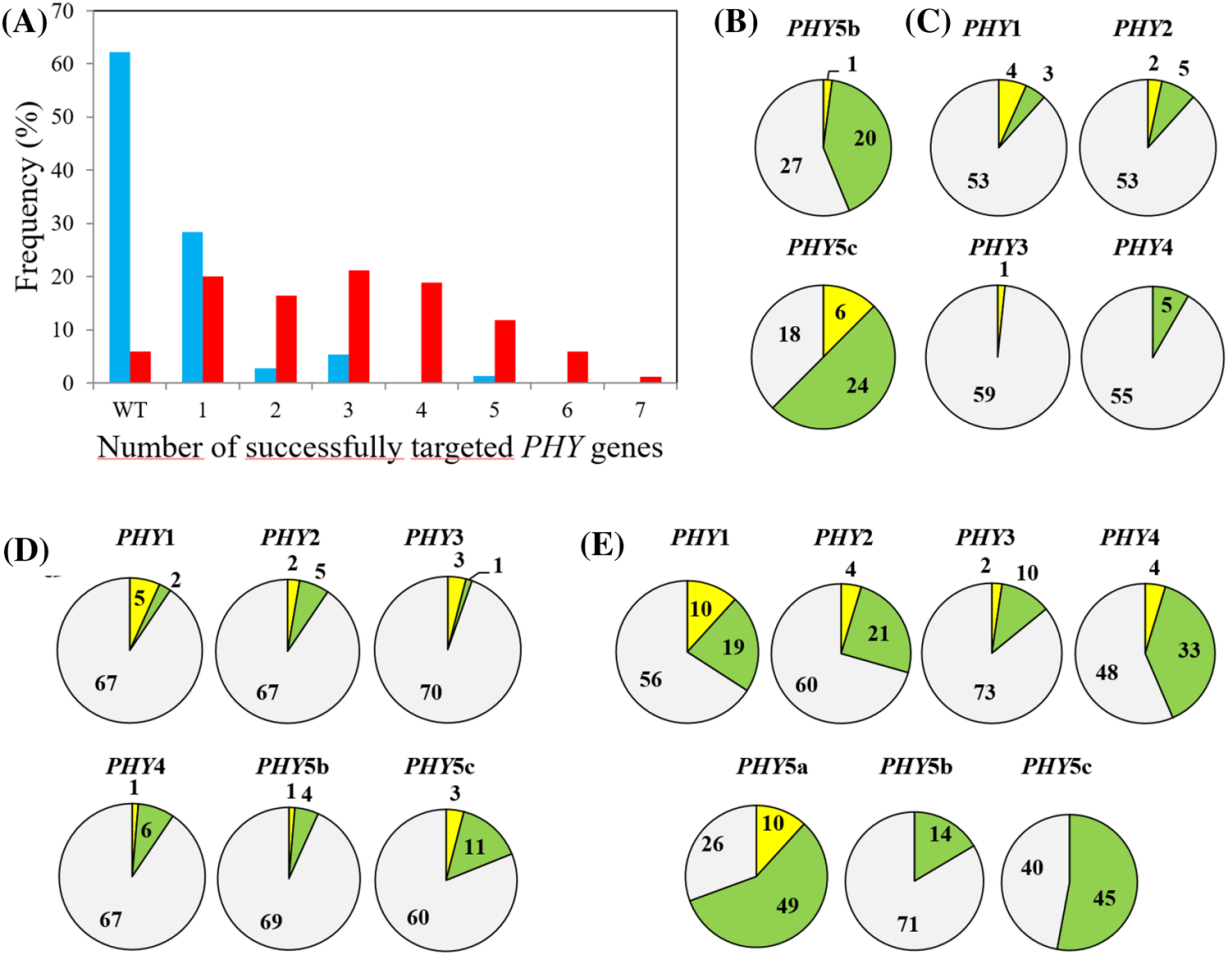
Cas9-mediated multiplex gene targeting in Physcomitrella. **a** Frequency distribution of single and multiple mutants obtained in transfections targeting 6 (blue, Table 1d) and 7 (red, Table 1e) *PHY* genes, the latter exploiting the pAL114 plasmid carrying both *Cas9* and the hygromycin resistance cassette. **b**, **c** Distributions of indels identified for each target in the transfections targeting 2 and 4 *PHY* genes (Table 1b, c). **d**, **e** Distributions of indels identified for each target in the transfections targeting 6 and 7 *PHY* genes (Table 1d, e), respectively. Deletions (green), insertions (yellow), wild-type loci (grey)

**Fig. 4:**
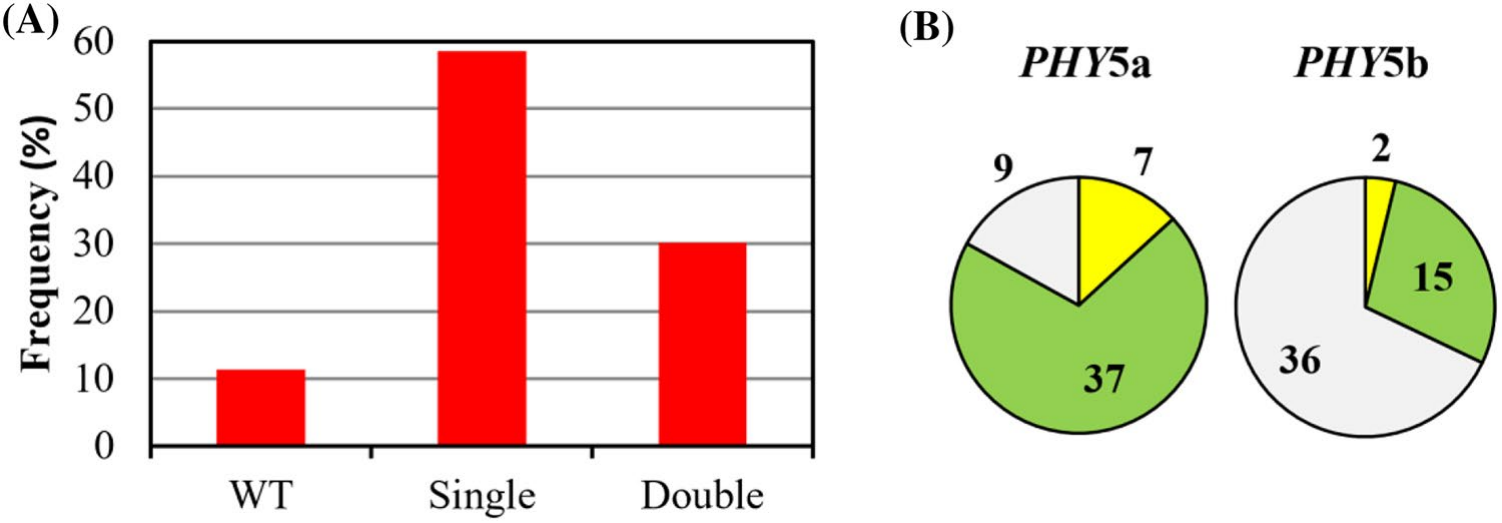
Retransfection of a Physcomitrella quintuple mutant targeting the two remaining wild-type genes *PHY5a* and *PHY5b* (Table 1f). **a** Frequency distribution of single and double mutants. **b** Distribution of deletion and insertion events. Deletions (green), insertions (yellow), wild-type loci (grey)

In preliminary experiments, we targeted phytochrome genes with Cas9 by transfecting Physcomitrella protoplasts with separate pAct-Cas9 and pBHRF hygromycin-resistance plasmids together with appropriate sgRNA constructs designed with the help of CRISPOR (Haeussler et al. 2016) according to established procedures (Ermert et al. 2019; Lopez-Obando et al. 2016) (Table 1a–d). After transient selection with hygromycin, we extracted gDNA using CTAB then amplified the targeted regions by PCR allowing indels down to ± 1 bp to be identified in high-resolution PAGE (see 'Materials and Methods') prior to sequencing. We thereby successfully targeted the sibling genes *PHY2* & *-4* (Table 1a) and *PHY5b* & *-c* (Table 1b), obtaining several double KOs in each case, whereas targeting *PHY1-4* (Table 1c) we identified two triple KOs (*phy1 phy2 phy4*) alongside double and single mutants but no quadruple mutant. We also targeted *PHY1-4* with *PHY5b & -c* (Table 1d) obtaining one quintuple KO mutant (*phy1 phy2 phy3 phy4 phy5c*) along with several lower-order mutants. Subsequently, we created the pAL114 plasmid carrying both Cas9 and the *35S*::hygromycin resistance cassette. This not only allowed us to increase the concentration of sgRNA plasmids in the mixtures, it linked the subsequent *loss* of transient hygromycin resistance with that of Cas9. With this improved system we targeted all 7 phytochrome genes in a single transfection (Table 1e), successfully generating a septuple along with several sextuple and numerous lower-order multiplex mutants. This was associated with a dramatically improved efficiency relative to the earlier sixfold targeting transfection and indeed most other experiments (Table 1; Fig. 3). We also generated a septuple mutant (Table 1f) by targeting the remaining wild-type *PHY5a* and *PHY5b* genes in a quintuple KO mutant generated earlier (Table 1d). For this we used the pSCOE-fcoCas9 plasmid containing a nptII cassette for kanamycin selection, avoiding the delay associated with waiting for the transient hygromycin resistance to be lost (see 'Materials and Methods'). We thereby obtained several sextuple and septuple KOs (Fig. 4).

### sgRNAs targeting efficiency

Cas9-mediated gene editing efficiency critically depends on the characteristics of the sgRNAs used. The PCR screening allowed us to compare the efficiencies of the different sgRNA constructs in the multiplex transfections (Table 1b–e, Fig. 3b–e). Although the sgRNAs used induced both insertions and deletions, deletions were more abundant (Supplementary Information, Table S1), as seen in previous studies (Collonnier et al. 2017). The relative efficiency of each sgRNA was consistent throughout, differences between experiments arising from the different plasmid concentrations used in each case (Table 1b–d) and in particular from the improved pAL114-based transfection procedure (Table 1e, f).

### Alternative approaches to Cas9-mediated knock-out

In order to investigate how CRISPR methods might be exploited most effectively in Physcomitrella*,* we evaluated the efficiency of different genome editing approaches targeting the *PHY4* locus. Thus, we investigated the significance of target spacing in dual-cut transfections by excising either a ~ 480 bp fragment between the 5′ UTR and the *PHY4* first exon or the entire ~ 6 kbp locus (Fig. 1a and b, respectively). In both cases the pAct-Cas9 and pBHRF plasmids together with two appropriate sgRNAs were employed to induce DSBs for repair through NHEJ. Surprisingly, the large deletion was consistently ~ fivefold more efficient than the smaller one (25% vs. 5.1%; Table 2).

We also tested the efficiency of HDR-enhanced gene excision of the *PHY4* locus with or without a stably selectable marker between the gene-homologous regions (Fig. 1c and d, respectively; Table 3). With marker insertion 22% of selected lines showed the desired mutation, and even without selection we found one excision event in 63 lines tested.

### Abrogation of gravitropism in light is mediated by phytochrome 5a

**Fig. 5:**
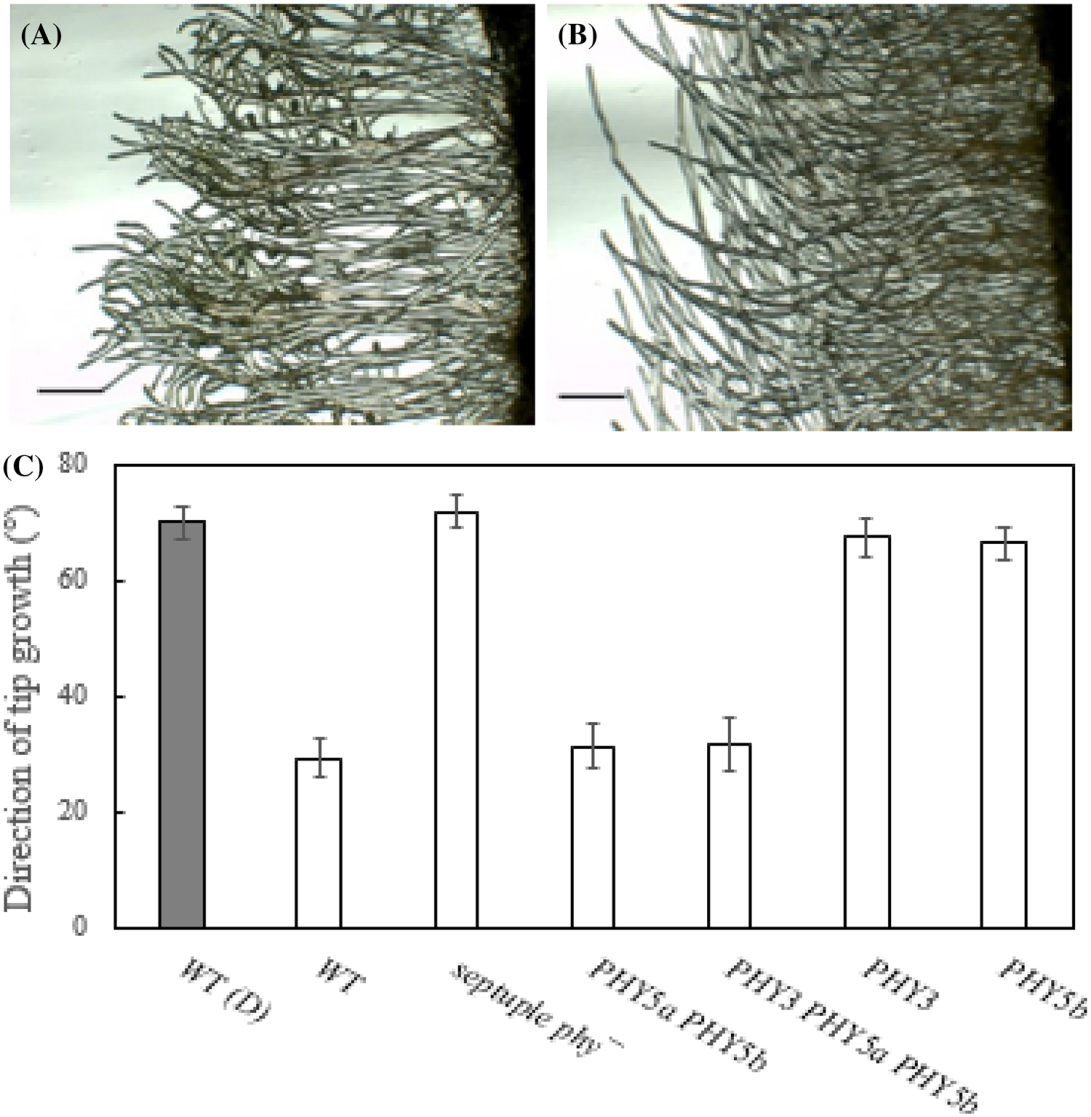
Phytochrome-mediated abrogation of gravitropism in Physcomitrella. **a** Wild type and **b** septuple KO lines after reorientation in red light. Scale bars: 1.5 mm. **c** Tip growth direction (mean ± SE) of WT in darkness and of WT and five mutant lines in red light (upper case refers to wild-type genes still present), namely *phy1 phy2 phy3 phy4 phy5a phy5b phy5c (the septuple knockout), phy1 phy2 phy3 phy4 PHY5a PHY5b phy5c, phy1 phy2 PHY3 phy4 PHY5a PHY5b phy5c, phy1 phy2 PHY3 phy4 phy5a phy5b phy5c* and *phy1 phy2 phy3 phy4 phy5a PHY5b phy5c*, respectively

In Physcomitrella, as in seed plants, the photoactivated phytochrome state, Pfr, suppresses gravitropism (Jenkins et al. 1986). We used the phytochrome mutant lines to identify the phytochrome/s responsible. Protonemata were initially grown on vertically-orientated Petri-dishes in darkness. Thereafter, the plates were rotated by 90° with respect to the gravity vector and irradiated with red light for 3 days (see 'Materials and Methods'). The strong negative gravitropism shown by the WT in darkness was inhibited by light, whereas the septuple null lines remained strongly gravitropic (Fig. 5). The responses of lower order multiplex KO lines indicated that gravitropic abrogation is primarily correlated with phy5a.

## Discussion

We carried out multiplex CRISPR-Cas9 mutagenesis of the *PHYTOCHROME* gene family in Physcomitrella, aiming to generate single and multiple KOs for functional genetics studies. We expanded the CRISPR toolkit itself by creating an improved transient selection/Cas9 plasmid as well as developing efficient screening procedures prior to sequencing, both of which contributed significantly to the success of the work. We also assessed the efficiencies of various Cas9-based KO strategies in targeting *PHY4* to excise either a small fragment or the entire coding region with or without HDR.

We improved the protoplast-transfection-based CRISPR system by placing the *HPT1* hygromycin resistance gene on the same plasmid as *Cas9* (pAL114). This reduced the number of plasmids in the mixture, allowing the concentration of sgRNA constructs to be increased, a factor that we consistently found to be important (see Table 1b–d and Fig. 2b–d). An additional aspect is that pAL114 provides better security that *Cas9* has not inserted into the genome. In these procedures, antibiotic selection is applied only transiently to eliminate cells that have not taken up DNA. After that, selection is removed, usually accompanied by loss of resistance—and thus now of Cas9 too—over the following weeks. The benefits of this new tool were especially evident when targeting all 7 *PHY* genes simultaneously (Table 1e). Our results (Fig. 3a) clearly show more efficient high-order multiplex targeting using pAL114 than with Cas9 and the antibiotic resistance gene on separate plasmids. With the improved method, ~ 60% of protoplasts still viable following the transfection procedure show transient resistance to hygromycin and thus have taken up DNA.

We observed that multiple *PHY* gene mutations occurred much more frequently than expected from the incidence of individual mutations. For example, in the sixfold targeting experiment (Table 1d), the projected probability of any quintuple mutant was about 10^–5^, whereas we found one such mutant in only 74 lines investigated. More convincingly, in the seven-fold targeting experiment using pAL114 (Table 1e), the projected probabilities of sextuple and septuple mutants were about 0.41% and 0.16%, respectively, whereas we obtained 5 and 1, respectively, in only 85 lines. A similar effect was seen in earlier work (Lopez-Obando et al. 2016). The high incidence of multiple mutations might arise from physiological differences between individual protoplasts regarding the effectiveness either of Cas9 in inducing DSB's or of the NHEJ machinery in repairing them, irrespective of the number of sgRNA plasmids present. Thereby, certain cells show much stronger mutagenesis for all targets than others, analogous to the phenomenon of the super-spreader in disease transmission (Lloyd-Smith et al. 2005).

The *PHY5b* and *PHY3* sgRNA constructs were least efficient whereas *PHY5a* and *PHY5c* sgRNA constructs were most efficient in both transfections targeting 6 and 7 PHY genes (Fig. 2d, e). Low CG content (< 25%) within the first 10 bp of the sgRNA (distal to the PAM) has been shown to reduce editing activity significantly (Labuhn et al. 2018). However, the PAM-distal CG content for all the sgRNA constructs in our study was > 30% (indeed, the *PHY3*-sgRNA was one of the highest at 60%), thus this character cannot explain the lower efficiency observed (Supplementary Information, Table S2). The constructs also bear none of the sequence motifs such as TT or GCC that commonly reduce CRISPR editing efficiency. On the contrary, the *PHY5c*-sgRNA carried the low efficiency TT-motif near its PAM sequence yet was particularly effective in our experiments. Quite possibly, the factors influencing CRISPR efficiency are different in Physcomitrella from those in human and mouse upon which the usual parameters are based (Graf et al. 2019). On the other hand, we found that the frameshift ratio for each sgRNA predicted by CRISPOR generally corresponded closely to our sequencing results except for the *PHY5b* construct which produced more in-frame mutations than expected (Supplementary Information, Table S1). Frameshift ratio is calculated based on the Lindel algorithm that uses data from mutational events in a human cell line and exploits the bias of NHEJ outcomes towards microhomology mediated events (Chen et al. 2019). The good correspondence observed here is in line with a similar bias observed in Physcomitrella towards microhomology mediated repair of the CRISPR-Cas9 mediated double strand breaks (Collonnier et al. 2017; Mara et al. 2019).

Mallet et al. recently described a cloning method in which ~ 50 bp oligonucleotide pairs corresponding to the protospacer are inserted into a pre-existing Cas9 sgRNA scaffold using Gateway recombination (Mallett et al. 2019). If generally applicable, this procedure would offer an alternative to sgRNA gene synthesis, reducing costs significantly. The authors also reported successful use of multisite Gateway to clone up to four protospacer sequences into a single plasmid, thereby targeting four genes. Thus, using three such plasmids it would theoretically be possible to knock out a dozen genes in one transfection experiment. This elegant approach might have significant advantages in multiplex targeting, albeit at the expense of flexibility in analysing the functions of individual genes. The Cpf1 (Cas12a) type V (Makarova et al. 2015) programmable endonuclease might be even more effective in gene targeting than Cas9, having been shown to generate threefold multiplex mutations with high efficiency in Physcomitrella (Pu et al. 2019). According to that study, Cpf1 generates large (− 8 to − 33 bp) deletions rather than the small indels (+ 2 to − 2 bp) seen with Cas9 in our work and that of others. In the case of KO mutagenesis via frame-shift this difference is unimportant, but single (or double) codon deletions which can be useful in particular circumstances are thus unlikely with Cpf1.

Efficient detection of multiplex indels is challenging. The first critical step requires efficient extraction of genomic DNA. Most current gDNA extraction techniques are unsuitable for high-throughput approaches. Additionally, in the case of plants, the cell wall is a literal obstacle for most extraction procedures. We developed an efficient, reliable and inexpensive extraction method to obtain gDNA from just a few milligrams of fresh protonemata appropriate for 96-well microtitre plates without the need of mixer-mill or other specialized equipment. By degrading the cell wall enzymatically rather than breaking it mechanically, we were able to release the content of the cells into the medium. The gDNA was then extracted using CTAB according to conventional methods. The second critical step is to identify indels efficiently. Conventionally, this is achieved by sequencing PCR products spanning each targeted region in each transiently resistant line. In the present work, this would have entailed about 2000 PCR/sequencing procedures. Instead, we devised a PCR-based screening procedure to identify indels prior to sequencing. We amplified a short (~ 100 bp) region flanking each targeted site, paying particular attention to primer specificity in view of the sequence similarities of the 7 *PHY* genes, and analyzed the product on non-denaturing polyacrylamide gels providing a resolution of ± 1 bp, thus allowing all indels to be identified easily (Fig. 4). This mobility-based screen is particularly valuable for large-scale screening in multiplex mutagenesis, providing for efficient identification of higher order mutants. Although the mobility shift can often be judged to represent ± 1, 2 or 3 bp, allowing likely frame-shift mutations to be identified, we invariably sequenced the PCR product. Huge savings in cost, labour and time were achieved nevertheless. We were surprised to find that when using classical Taq DNA polymerase instead of the Phusion DNA polymerase mixture we invariably observed two bands with a mobility difference corresponding to 2 bp (Fig. 2). The longer product probably derives from the well-known addition of unpaired single bases to the 3′ ends of the products of polymerases lacking 3′ → 5′ exonuclease error checking activity (Clark 1988). It is intriguing that, although only about half of the products show reduced mobility, intermediate bands that would correspond to additions to only one of the two strands were never seen. Given that PCR is the single most commonly used method in molecular genetics over the past 30 years, it is quite remarkable that this effect seems not to have been reported earlier. In practice, the dual product can be useful as it allows precise calibration of the electrophoretic mobility in terms of bp lost or gained.

It is possible to introduce larger deletions if a gene is targeted with two sgRNAs (Ermert et al. 2019). Furthermore, the resulting deletion can then be repaired by the HDR pathway when a donor DNA is provided, allowing the whole gene or a section of it to be replaced by another sequence. Widely spaced sgRNAs allow extensive enhancer regions or even gene clusters to be removed or replaced. Very large deletions are possible in principle, 245 kbp deletions having been detected by PCR in rice protoplasts (Zhou et al. 2014). CRISPR-Cas9-mediated fragment deletions of 4.5, 10 and 0.9 kb have been reported in regenerated soybean, rice and *Arabidopsis,* respectively (Cai et al. 2018; Wang et al. 2017; Zhao et al. 2016). ~ 3 kbp deletions have been reported in Physcomitrella (Nomura et al. 2016), extended to ~ 6 kbp for the *PHY4* locus in this study. Surprisingly, we found that the ~ 6 kbp excision was five times *more* efficient than a much shorter (~ 480 bp) fragment using NHEJ (Table 2). Although this might be the result of large differences in the efficiencies of the sgRNA constructs, the sgRNAs used were predicted to be similarly efficient. Whatever the reason for the difference, our results suggest that large deletions mediated by Cas9 might be facile in Physcomitrella.

CRISPR-mediated gene targeting using HDR is challenging in seed plants, with most attempts reporting low success rates (Gil-Humanes et al. 2017; Peng et al. 2020; Shi et al. 2017). The exceptionally high rate of HR in Physcomitrella appears to facilitate Cas9-mediated KO and in particular HDR-mediated gene replacement. We show that CRISPR-induced HDR is highly proficient in removing the whole ~ 6kbp *PHY4* locus with the insertion of an antibiotic cassette (Table 3a). This efficiency is similar to that with NHEJ in our experiments (Table 2b), and nearly tenfold better than CRISPR-mediated single-cut gene targeting with a homologous repair sequence in *Arabidopsis* (Peng et al. 2020). Probably on account of the efficient HR in Physcomitrella we even obtained a single HDR mutant in 63 tested lines without using an insertional marker (Table 3b).

The likely involvement of the MMEJ repair pathway for cleavage products generated through CRISPR editing has been observed in a variety of human cell types (Bae et al. 2014). Our data for Physcomitrella is in line with this, as in previous studies (Mara et al. 2019; Seol et al. 2018). Efficient MH-mediated repair in Physcomitrella might be connected to its potentially particular handling of the DSBs linked also to its unique HR-proficiency, when compared to the CRISPR-induced mutation pattern in *Arabidopsis*. Indeed, we observed that indels of 1 or 2 bp (which are not repaired with the help of microhomologies) appear much less frequently compared to *Arabidopsis* (Pauwels et al. 2018).

Off-target activity is a perennial problem in gene targeting. We assayed this in the six six- and sevenfold mutants by analyzing all eight of the off-target sites predicted by CRISPOR (Supplementary Information, Table S3). None was found. Although of course other off-target mutations might have arisen, most events of this kind in diverse species involve closely similar sequences. As no off-target mutations were found using similar methods in other Physcomitrella studies (Lopez-Obando et al. 2016; Nomura et al. 2016), we consider that targeting is very specific in this system.

We created this mutant collection to help clarify phytochrome functions in Phydcomitrella, in particular regarding phototropism (Hughes 2013; Mittmann et al. 2004). As proof of principle, we investigated the light-dependent abrogation of gravitropism. Most plants show strong negative gravitropism in darkness, a response that helps them escape from the darkness of the soil, whereas in the light the response is repressed following the formation of Pfr. Accordingly, light repression of gravitropism is weakened in mutants either lacking the appropriate functional phytochrome/s or in which the Pfr signal is blocked. For example, abrogation of gravitropism in *Ceratodon* filaments in light is lost in heme oxygenase null mutants unable to synthesize the phytochrome chromophore (Lamparter et al. 1996). In harmony with that result, here we found that septuple *phy*^*−*^ null mutants remained gravitropic in light (Fig. 5b). We then tested the abrogation phenotype in different lower-order KO lines, finding that phy5a is the photoreceptor primarily involved (Fig. 5c). As the approach thus seems to be successful, the mutant library provides a useful resource for the phytochrome community.

In summary, our optimized transfection and targeting methods alongside efficient gDNA extraction, pre-screening for indels via PCR and high-resolution PAGE gels proved effective in CRISPR-based multiplex gene editing in Physcomitrella. We demonstrated the efficacy of the methods by inactivating all seven *PHYTOCHROME* genes and showing the loss of a classical phytochrome-mediated response to light in septuple *PHY* mutants and with the help of lower-order mutants were able to identify the specific phytochromes primarily responsible. We thus expect that our mutant collection will be useful in phytochrome research and that, in general, CRISPR-based genome editing in Physcomitrella will provide unique functional insights into plant biology through efficient genome editing.

## Supplementary information

Below is the link to the electronic supplementary material.

Supplementary material 1 (XLSX 51 kb)

Supplementary material 2 (DOCX 22 kb)

## Data Availability

The plasmids and Physcomitrella mutant library described here are available to the community.
