# Supplementary material for: Multiplex CRISPR-Cas9 mutagenesis of the phytochrome gene family in Physcomitrium (Physcomitrella) patens
Source: Plant Mol Biol. 2020 Dec 21;107(4-5):327–36. doi: 10.1007/s11103-020-01103-x (PMC8648701; doi:10.1007/s11103-020-01103-x)
Supplement: Supplementary file 2 — Supplementary material 2 (DOCX 22 kb) [file 11103_2020_1103_MOESM2_ESM.docx]

SUPPLEMENTARY INFORMATION

**Table S1 Lines produced in the multiplex *PHY* mutagenesis using single sgRNA's (Excel file)**

**Table S2 Target genes and crRNA sequences for multiplex gene editing (see Table 1)**

| Gene^a^ | crRNA sequence | Specificity Score^b^ (%) |
| --- | --- | --- |
| *Pp3c25_2610V1.1 (PHY1)* | GACCCTCACACTATGCGCCT **TGG** | 100 |
| *Pp3c16_20280V1.1 (PHY2)* | GTCAAAGCATAGTGTCCGGG **TGG** | 100 |
| *Pp3c16_18760V3.1 (PHY3)* | AGTCGAAGGCCCACAGCGTG **AGG** | 100 |
| *Pp3c27_7830V3.3 (PHY4)* | GGCAGCCTATTAGTCTGGCC **GGG** | 100 |
| *Pp3c3_23790V1.1 (PHY5a)* | CACAAGCTGTACCGAGTGTG **GGG** | 100 |
| *Pp3c12_9240V3.1 (PHY5b)* | ACTTCCTTTAGACCGACACT **TGG** | 100 |
| *Pp3c4_15350V3.1 (PHY5c)* | GCGCGGCTACAATCCCTTCC **CGG** | 99 |

^a^ Identifier and name (in brackets) of genes from Phytozome V11 - *P. patens* genome.

^b^ Specificity score data from CRISPOR (Hsu *et al.*, 2013).

**Table S3 Targeted genes and CRISPOR-predicted off-target sequences**

| **On-target gene** | **Predicted off-target sequence^a^** | **Off-target annotation** |
| --- | --- | --- |
| ***PHY1*** | GACTCTCACAGTCTGCCCCT **CGG**  GACTCTCACAGTATGGCCCT **CGG**  GACTCTCACACTCTGGCCCT **TGG** | Off-T1: Non-coding DNA region  ^*^Off-T2: Non-coding DNA region  Off-T3: Scaffold38 |
| ***PHY2*** | GTCAAAGCATTGTGACAGGT **CGG**  GTCACGGCTTAGTTTCCGGG **AGG**  GTCAAAGCAGAGGTTCCGGT **AGG** | Off-T1: Non-coding DNA region  ^*^Off-T2: *Pp3c1_35220*  ^*^Off-T3: *Pp3c14_9939* |
| ***PHY3*** | AGTCCAAGGCGCATAGTGTG **AGG** | Off-T1: Exon of *Pp3c25_2610* |
| ***PHY5a*** | AACAAGCTGAACTGAGTGAG **GGG**  CAAAAGATATACCTAGTGTG **TGG** | ^*^Off-T1: *Pp3c20_21890*  Off-T2: Non-coding DNA region |
| ***PHY5b*** | ACTTCCTGCTGACCCACACT **GGG**  ACTTCCTGTTGGCCCACACT **TGG**  ACTTCCTTTAGCCAGCCATT **TGG** | ^*^Off-T1: Exon of *Pp3c27_7830*  ^*^Off-T2: 5’ UTR of *Pp3c27_7830*  ^*^Off-T3: 5’ UTR of *Pp3c7_6260* |
| ***PHY5c*** | TCGCGGTTGCAATCCCTTCC **CGG** | ^*^Off-T1: Exon of *Pp3c12_9240* |

^a^ Mismatch of off-target sequence with respect to the crRNA sequence is shown in yellow.

^*^ Off-targets examined.

**Table S4 List of primers used in the multiplex *PHY* gene editing via single-cut**

| **Name** | **Primer sequence** | **Description** |
| --- | --- | --- |
| FST5 FW | CCCAAGAAGACATACTCCTCAAC | *(Pp.PHY1)*PCR screening |
| FST6 RV | GAAGTGCTGCATCTGCGGTT | *(Pp.PHY1)*PCR screening |
| FST7 FW | GTCAGGGAGTTCTGTGAAGTCA | (*Pp.PHY2)* PCR screening |
| FST8 RV | TCCAGATTCTTCGTACACAGCC | (*Pp.PHY2)* PCR screening |
| FST11 FW | CTTACTCCTCCACAAGTTCG | *(Pp.PHY3)* PCR screening |
| FST12 RV | TTTCAAACACAGCATGAAGG | *(Pp.PHY3)* PCR screening |
| FST13 FW | AAAGTGGTTCAGGACAAGGAT | (*Pp.PHY4)* PCR screening |
| FST14 RV | GAGTTCATGTTGCCCATGTAT | (*Pp.PHY4)* PCR screening |
| ST15 FW | GCTTACAGCGAGAATGCTCT | (*Pp.PHY5a)* PCR screening |
| ST16 RV | CAATGATCTCGAGTCGGTTCC | (*Pp.PHY5a)* PCR screening |
| FST17 FW | ACAGTGAGAATGCGCCCGAA | (*Pp.PHY5b)* PCR screening |
| FST18 RV | CATCGGTTCCGATTCCGAGG | (*Pp.PHY5b)* PCR screening |
| FST19 FW | ATTCCCACAAATTGGCAGCT | (*Pp.PHY5c)* PCR screening |
| FST20 RV | CCACAACTGCATCACACAGT | (*Pp.PHY5c)* PCR screening |
| FST37 FW | GCTACCGGATCTCGGAGTTG | (*Pp.PHY1) S*equencing reaction |
| FST38 RV | GAGAGGCGACACTTGAAGCT | (*Pp.PHY1)* Sequencing reaction |
| FST23 FW | GAGCTGCCTGGTTTAGTGAC | (*Pp.PHY2)* Sequencing reaction |
| FST24 RV | TCAGCATAGTCACATCCACA | (*Pp.PHY2)* Sequencing reaction |
| FST35 FW | ACACTCGGCTACAGTTCTGC | (*Pp.PHY3)* Sequencing reaction |
| FST36 RV | GTCCCGATTCCTAGCGTGTTTAT | (*Pp.PHY3)* Sequencing reaction |
| FST26 FW | ATGCCGTTGTGTCCTCCGCA | (*Pp.PHY4)* Sequencing reaction |
| FST27 RV | GTGTGATTCCCAAAAGCCAAAACCT | (*Pp.PHY4)* Sequencing reaction |
| ST33 FW | GAATCAAACGACTCTGGGGA | (*Pp.PHY5a)* Sequencing reaction |
| ST34 RV | CTGTTGCAGGATAATGTAGCC | (*Pp.PHY5a)* Sequencing reaction |
| FST31 FW | ACACCTCATAGCGGTTTTGG | (*Pp.PHY5b)* Sequencing reaction |
| FST32 RV | ACTTCCTCCACAACTGCGTC | (*Pp.PHY5b)* Sequencing reaction |
| FST29 FW | GTCAAGCTGCCAAAGCAACC | (*Pp.PHY5c)* Sequencing reaction |
| FST30 RV | CAAGAGTGATTGGCTGACGC | (*Pp.PHY5c)* Sequencing reaction |
| PHY1_OFFT2 FW | GCTTGCCGACGGATCATCTT | Off-target analysis |
| PHY1_OFFT2 RV | TCCTCAATAGCGGCACCAAAA | Off-target analysis |
| PHY2_OFFT2 FW | AAGCAGACAAGGCTGATCCC | Off-target analysis |
| PHY2_OFFT2 RV | TAGTCATCATCAGCGCGGTC | Off-target analysis |
| PHY2_OFFT3 FW | CTTGGGACGGAGAGAAGGTG | Off-target analysis |
| PHY2_OFFT3 RV | ACTGCTTTCCTGCAAATCGC | Off-target analysis |
| PHY5a_OFFT1 FW | GGTAAACGCGCGCAAAAGAT | Off-target analysis |
| PHY5a_OFFT1 RV | CACTCTTCTGGCGGTACGTC | Off-target analysis |
| PHY5b_OFFT1 FW | GAATGCGCCAGAGATGCTTG | Off-target analysis |
| PHY5b_OFFT1 RV | AAAGGAGGCCAATGTCTCCG | Off-target analysis |
| PHY5b_OFFT2 FW | TAAGGGCGTGTCGGATTGTT | Off-target analysis |
| PHY5b_OFFT2 RV | AAGACCTCACCGTGTTCGTC | Off-target analysis |
| PHY5b_OFFT3 FW | AGCTTCCTCACCTCGCCTTC | Off-target analysis |
| PHY5b_OFFT3 RV | CCGAACCCTGTCCCAAATGT | Off-target analysis |
